# Chemical Vapor Deposition of Organic-Inorganic Bismuth-Based Perovskite Films for Solar Cell Application

**DOI:** 10.1038/s41598-019-46199-4

**Published:** 2019-07-05

**Authors:** S. Sanders, D. Stümmler, P. Pfeiffer, N. Ackermann, G. Simkus, M. Heuken, P. K. Baumann, A. Vescan, H. Kalisch

**Affiliations:** 1 Compound Semiconductor Technology, RWTH Aachen University, Sommerfeldstr. 18, 52074 Aachen, Germany; 2 AIXTRON SE, Dornkaulstr. 2, 52134 Herzogenrath, Germany; 3 APEVA SE, Dornkaulstr. 2, 52134 Herzogenrath, Germany

**Keywords:** Solar cells, Electronic devices, Design, synthesis and processing, Solar cells

## Abstract

Perovskite solar cells have shown a rapid increase of performance and overcome the threshold of 20% power conversion efficiency (PCE). The main issues hampering commercialization are the lack of deposition methods for large areas, missing long-term device stability and the toxicity of the commonly used Pb-based compounds. In this work, we present a novel chemical vapor deposition (CVD) process for Pb-free air-stable methylammonium bismuth iodide (MBI) layers, which enables large-area production employing close-coupled showerhead technology. We demonstrate the influence of precursor rates on the layer morphology as well as on the optical and crystallographic properties. The impact of substrate temperature and layer thickness on the morphology of MBI crystallites is discussed. We obtain smooth layers with lateral crystallite sizes up to 500 nm. Moreover, the application of CVD-processed MBI layers in non-inverted perovskite solar cells is presented.

## Introduction

Organic-inorganic halide perovskites offer a unique combination of properties including long carrier lifetimes^1,2^ and high absorption coefficients^1,3^, rendering these materials promising candidates for solar cell application. Recently, Pb-based perovskite solar cells have overcome the threshold of 20% power conversion efficiency (PCE) for single-junction devices^4–9^. Perovskite-silicon tandem solar cells were demonstrated with a PCE up to 27.3%^10^. The main issues hampering commercialization of this technology are the toxicity of Pb, a lack of deposition methods for large areas and limited long-term cell stability. To avoid the toxic and heavily regulated Pb, alternative perovskite materials based on Sn or Bi cations were investigated^11–15^. While Sn-based perovskites suffer from oxidation of Sn^2+^ to Sn^4+^, leading to an instant degradation of photovoltaic (PV) performance^16–18^, Bi-based perovskites exhibit superior air stability^19^. Exposing these layers to ambient air induces the formation of a thin Bi_2_O_3_ or BiOI film on the surface which protects the bulk^19^.

Spin-coating is the most common deposition method for perovskites. However, processing from liquid phase suffers from low reproducibility, limited substrate sizes, and in the case of methylammonium bismuth iodide (MBI), also poor coverage^20–22^. In contrast, chemical vapor deposition (CVD) offers improved process control, higher reproducibility and no fundamental limitations of substrate size. Thus, pure gas phase processes as well as combinations of solution and gas phase processes were investigated, leading to more efficient perovskite solar cells compared to those fully processed from solution^13,14,19^. Hoye *et al*. demonstrated a vapor-assisted conversion of spin-coated BiI_3_ to MBI by exposure to methylammonium iodide (MAI) in a saturated atmosphere. Those films exhibit improved lifetime of photo-excited species and higher crystallinity compared to solution-processed perovskites^19^. With an identical deposition route, the highest PCE reported for Bi-based perovskite solar cells of 3.17% is reached, due to the reduction of metal defect sites, enhanced crystallization and improved surface coverage^13^. Zhang *et al*. demonstrated dense MBI layers fabricated by a dry two-step deposition process. Via thermal evaporation, a BiI_3_ layer is formed and afterwards converted to MBI in an MAI-saturated atmosphere, exhibiting long-term stability over 15 weeks (stored in nitrogen atmosphere, measured in ambient air once per week) and a PCE of 1.64%^14^. However, either solvents or high-vacuum processes are required to deposit perovskite films with these methods.

Here, we demonstrate a solvent-free low-vacuum process using an in-house developed CVD reactor to deposit the precursors MAI and BiI_3_ simultaneously. This is to our knowledge the first showerhead-based deposition tool for perovskites, offering precise process control options and paving the way for large-area production. We analyze the influence of the ratio of the supplied precursors and of the substrate temperature on the layer crystallinity, morphology and optical properties. Finally, the implementation of MBI layers in standard non-inverted perovskite solar cells is demonstrated.

## Methods

Unless otherwise noted, materials were purchased from Sigma-Aldrich and used without further purification. The deposition of the perovskite films was studied on a typical solar cell anode stack on glass consisting of fluorine-doped Sn oxide (FTO)/compact TiO_2_ (c-TiO_2_)/mesoporous TiO_2_ (mp-TiO_2_). FTO-on-glass substrates (VisionTek Systems, 6–9 Ω/sq, 2.5 cm × 2.5 cm) were cleaned sequentially with dimethyl sulfoxide (DMSO), acetone and isopropyl alcohol, followed by rinsing in deionized water (DI) and drying with nitrogen. All following process steps (except for TiO_2_ oxidation/annealing in a muffle furnace) were carried out either under vacuum or purified nitrogen atmosphere. For the c-TiO_2_ layer, 15 nm of Ti were evaporated (rate 0.2 nm/s) using an e-beam evaporator under high vacuum (p < 10^−6^ hPa). For mp-TiO_2_, TiO_2_ paste (99% anatase, average particle size 20 nm) was dissolved in 2-methoxyethanol with 3.3 wt% and spin-coated on top of the Ti film at 1000 rpm for 30 s. Oxidation and annealing were performed for 5 min at 130 °C on a hot plate and for 30 min in a muffle furnace at 500 °C.

**Figure 1:**
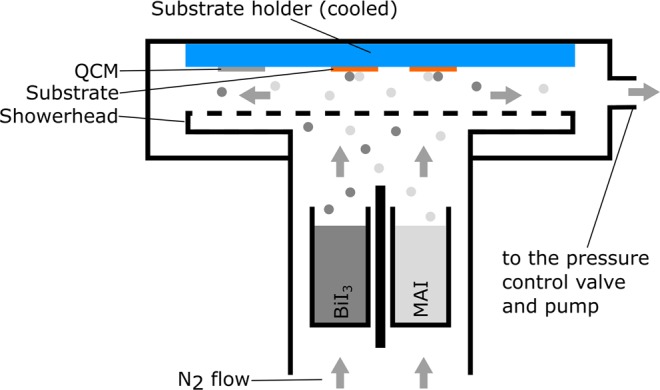
Schematic illustration of the deposition tool. The N_2_ flow was set to 500 sccm and the base pressure to 10 hPa.

A schematic of the CVD tool is depicted in Fig. 1 (left). The base pressure was set to 10 hPa (10 mbar) and the N_2_ flow to 500 sccm (standard cubic centimeter per minute). The evaporation source consists of two crucibles for BiI_3_ and MAI, which are separated by a shield to avoid cross contamination. The BiI_3_ crucible was heated up to 240–270 °C and the MAI crucible to 180–200 °C, depending on the desired MAI/BiI_3_ ratio and the current filling levels of the crucibles. The showerhead was kept at 260 °C and disperses the gas mixture of carrier gas and sublimed molecules, enabling a homogeneous deposition on large-area substrates up to 108 cm² (12 cm × 9 cm). A quartz crystal microbalance (QCM) was used to determine the deposition rate. The temperature of the substrate was adjusted by using a controlled water cooling circuit.

To fabricate perovskite solar cells, the hole-transport material 2,2′,7,7′-tetrakis[*N*,*N*-di(4-methoxyphenyl)amino]-9,9′-spirobifluorene (8 wt%, 99% purity) (Spiro-MeOTAD), lithium bis(trifluoromethanesulfonyl) (0.007 wt%, 99.95% purity) (Li-TFSI) and 4-*tert*-butylpyridine (0.01 wt%, 96% purity) were dissolved in acetonitrile and chlorobenzene (1:10 volume ratio). The solution was stirred overnight and spin-coated on top of the MBI layer at 2000 rpm for 30 s. For the top-contacts, 80 nm of Au were deposited using e-beam evaporation (0.2 nm/s) and shadow masks (circular, 19 mm², nine devices per substrate).

X-ray diffraction (XRD) 2θ scans with a fixed grazing angle of incidence were carried out using a Philips XPERT Pro tool. To evaluate the XRD patterns, background subtraction, 5-point Savitzky-Golay smoothing^23^ and normalization to the highest intensity were used. Morphological characterization of the deposited films was performed by using scanning electron microscopy (SEM, Zeiss Sigma). Absorption properties of the perovskite films were determined via a PerkinElmer LAMBDA 25 spectrophotometer. Illuminated I-V characteristics were measured using a Keithley 2400 source meter and a Newport S94023A-SR1 solar simulator (AM1.5 at 100 mW/cm^2^).

## Results and Discussion

**Figure 2:**
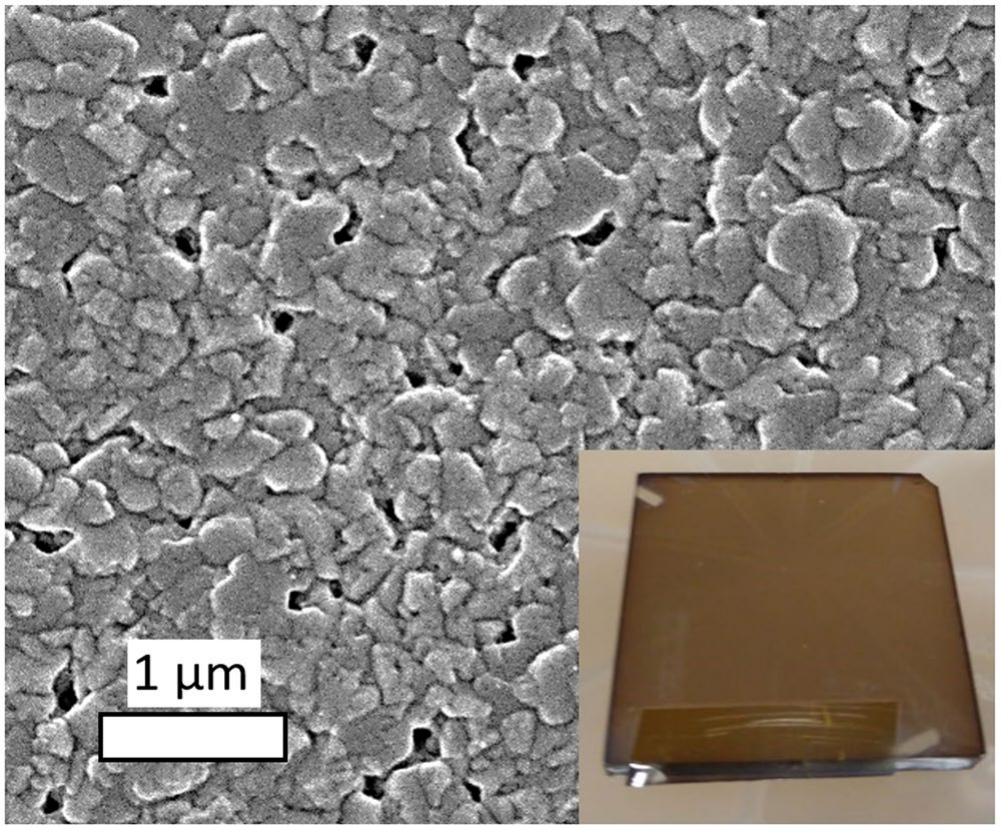
SEM image of a pure BiI_3_ film (layer thickness 175 nm, temperature of the substrate 88 °C, BiI_3_ rate 1.0 nm/min) on FTO/c-TiO_2_/mp-TiO_2_ substrates. The inset shows the macroscopic appearance (photography, 2.5 cm × 2.5 cm).

For this study, we deposited MBI films with different precursor ratios (controlled by the evaporation temperatures) and layers at different substrate temperatures in order to analyze the impact on film crystallinity and layer morphology. First, separate deposition experiments of MAI and BiI_3_ were conducted for QCM calibration. The morphology of a BiI_3_ film, processed at substrate temperature of 88 °C, is depicted in Fig. 2 (right). The dense film (layer thickness 175 nm, rate 1.0 nm/min) contains crystallites with an average grain size of around 200 nm. The MAI-only film was processed also using 88 °C substrate temperature and a rate of 3.0 nm/min.

To obtain stoichiometric MBI, we have to consider the different volumetric molecular densities (mol/cm³) of MAI (CH_3_NH_3_I) and BiI_3_. With respect to the molar weights M and densities d of both precursors (M_MAI_ = 159 g/mol, d_MAI_ $\approx$ 1 g/cm³ for organic matter^24^, M_BiI__3_ = 590 g/mol, d_BiI__3_ = 5.8 g/cm³^25^), a deposited-volume ratio (measured by the QCM rates) of 1.6 is required for equal molarity. The molecule MBI (MA_3_Bi_2_I_9_) contains of 3 methylammonium and 2 bismuth ions, leading to an ideal molar ratio of MAI and BiI_3_ precursor molecules of 1.5. As a result, a volume ratio (adjusted by the QCM rates) of 1.6 × 1.5 = 2.4 between MAI and BiI_3_ is required to deposit stoichiometric MBI. However, during simultaneous deposition of both precursors, the ideal ratio can deviate from the calculation due to the significantly larger vapor pressure of MAI^26^, the uncertainty in d_MAI_, chemical pre-reactions or sublimation.

**Figure 3:**
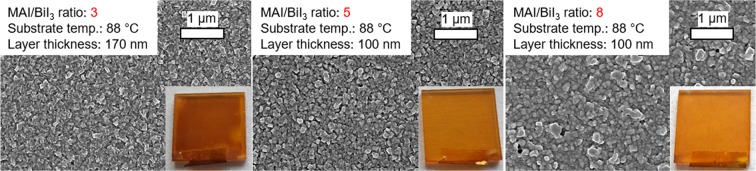
SEM images of MBI films deposited at 88 °C substrate temperature, a BiI_3_ rate of 0.5 nm/min and different MAI/BiI_3_ rate ratios of 3, 5 and 8. The insets show the macroscopic appearances (photography, 2.5 cm × 2.5 cm).

To deposit stoichiometric MBI, we varied the ratio of the QCM rates between MAI and BiI_3_ during film formation and analyzed its impact on the layer morphology, optical properties and crystal structure. The BiI_3_ rate was set to 0.5 nm/min, and the MAI rate was adjusted to obtain the ideal ratio between MAI and BiI_3_ rates. Previous experiments (not shown here) showed no MBI formation for MAI/BiI_3_ ratios ≤2 and thus the necessity of higher ratios. By employing a MAI/BiI_3_ ratio of 3 (close to the ideal ratio) and ratios of 5 and 8, we obtained the films shown in Fig. 3. As can be seen from the sample images in Fig. 3, for layers deposited with the lowest MAI rate (MAI/BiI_3_ ratio of 3), a darker film was obtained. This is a strong indication for residual BiI_3_, due to insufficient supply of MAI, and thus its incomplete conversion to MBI. The layers deposited with higher MAI/BiI_3_ ratios are of orange color, which is characteristic for MBI thin films. Consequently, an MAI/BiI_3_ ratio higher than the expected theoretical ratio of 2.4 is required, indicating a reduced MAI deposition most likely due to the instant sublimation of condensed (non-reacted) MAI throughout the process.

The average size of the crystallites is around 100 nm for layers with the two lower MAI/BiI_3_ ratios of 3 and 5. For the film processed with the highest MAI/BiI_3_ ratio of 8, the crystallite size varies in the range of 100–200 nm, exhibiting a rougher layer due to partially vertical growth. All substrates are fully covered by MBI, ensuring an effective separation between the titanium dioxide and the hole transport layer Spiro-MeOTAD, preventing shunting pathways in the perovskite solar cells. For that reason, these layers are superior for solar cell application, compared to those MBI films which are not entirely closed and provided by liquid-phase processing^22^.

**Figure 4:**
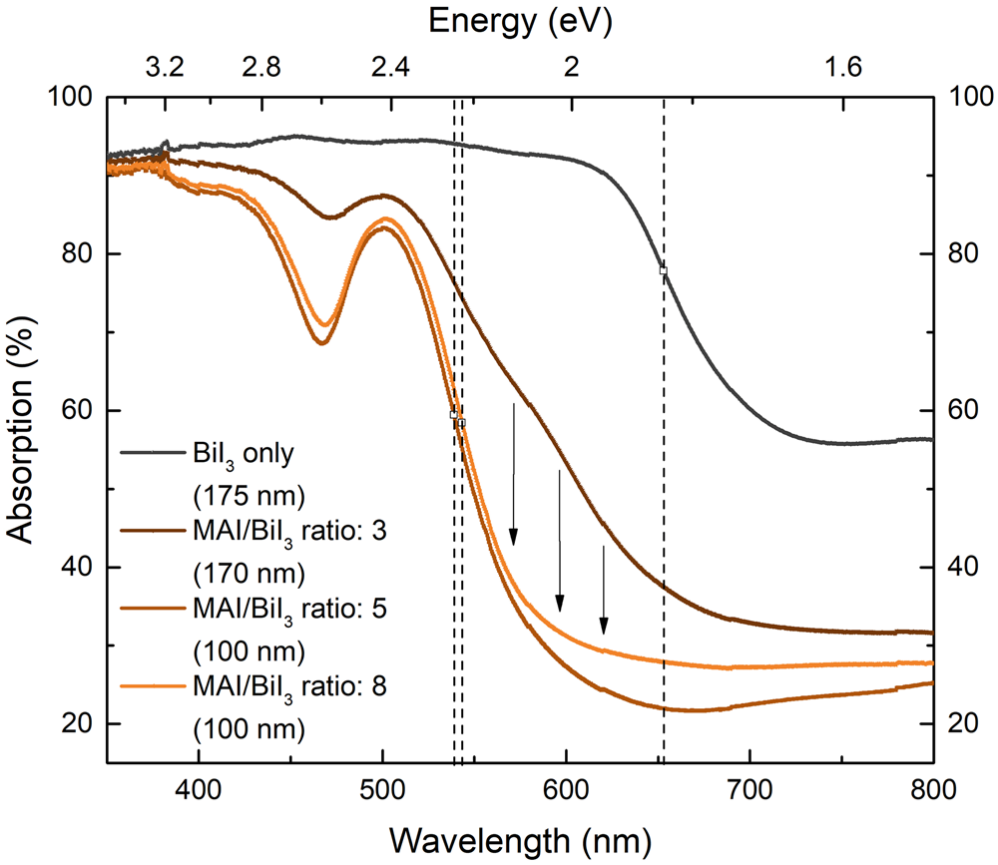
Absorption spectra of the BiI_3_-only film and MBI layers on FTO/c-TiO_2_/mp-TiO_2_ substrates deposited with a BiI_3_ rate of 0.5 nm/min, a substrate temperature of 88 °C and different MAI/BiI_3_ ratios. The interpolated absorption edges are exhibited by the dashed vertical lines. The arrows illustrate the drop of absorption in the region of 500 to 650 nm for MBI layers without residual BiI_3_.

The corresponding absorption spectra of the BiI_3_ sample and MBI films are depicted in Fig. 4. The BiI_3_-only layer (optical bandgap = 1.8 eV^27^) has a broad absorption spectrum from 430 to 620 nm, and the absorption edge can be interpolated to 652 nm (1.90 eV), corresponding to the optical bandgap of the material.

The absorption spectra of the MBI films exhibit a strong excitonic absorption peak at 502 nm (2.47 eV), derived from the electron transition of ^1^S_0_ to ^3^P_1_ from the trivalent Bi^3+^ in the [Bi_2_I_9_]^3−^ bioctahedrons^28^, which is a characteristic for MBI. The sample with a rate ratio of 3 does not show a steep edge on the lower-energy side at the excitonic peak (in the region of 500 to 650 nm) compared to those samples with higher MAI/BiI_3_ ratios. This is again a strong indicator of residual BiI_3_ in the MBI film^14^ and correlates to its darker color. The samples with higher MAI rate exhibit a steep edge at the excitonic peak, confirming the MAI amount to be sufficient for a complete conversion of BiI_3_ to MBI. Moreover, for the samples with MAI/BiI_3_ rate ratios of 5 and 8, the absorption edge of MBI can be interpolated to 539 nm (2.30 eV) and 543 nm (2.28 eV), respectively. These values represent the optical bandgap of MBI and are in good agreement with the literature value of 2.26 eV, derived from a Tauc Plot^14^. The sample with the largest MAI/BiI_3_ ratio of 8 shows higher absorption for wavelengths >575 nm compared to the sample with a ratio of 5. The reason for this sub-bandgap absorption can be crystallographic defects. These defects can create states in the bandgap, which absorb photons with sub-bandgap energy^29^. Thus, the sample with an MAI/BiI_3_ ratio of 5 shows the best optical properties.

**Figure 5:**
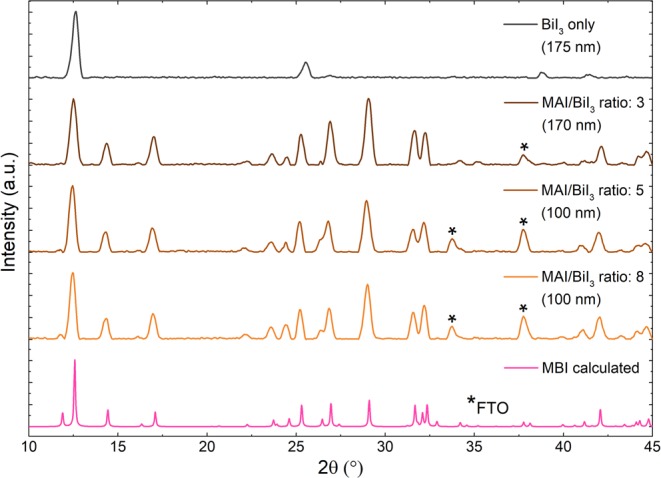
Calculated XRD pattern of MBI and XRD measurements of BiI_3_-only film and MBI layers deposited with different MAI/BiI_3_ ratios (3, 5 and 8), substrate temperature of 88 °C and a BiI_3_ rate of 0.5 nm/min.

The XRD spectra of the pure BiI_3_ film and the MBI layers (MAI/BiI_3_ ratios of 3, 5 and 8) are compared to a calculated XRD pattern of MBI^30^ (Fig. 5). For the BiI_3_-only film, the most significant peaks are located at 12.6°, 25.5° and 38.8°, which were also observed in literature^31^. The measured XRD peaks of CVD-processed MBI layers for all investigated MAI/BiI_3_ ratios match those of the calculation. Additional signals at 33.8° and 37.8° (marked with*) correspond to FTO. As expected, with decreasing MBI layer thickness, their relative intensities become more prominent. Despite the indication of excess BiI_3_ by previously discussed absorption measurements, the presence of residual BiI_3_ for the sample with the MAI/BiI_3_ ratio of 3 cannot be confirmed by XRD analysis. A possible reason can be that the main BiI_3_ and MBI peaks are located at the same angle of 12.6°, and the remaining BiI_3_ peaks exhibit insufficient intensities. For the sample with an excessive amount of MAI (MAI/BiI_3_ ratio of 8), no significant differences compared to the sample with an MAI/BiI_3_ ratio of 5 and thus, no characteristic MAI peaks (located at 2θ = 9°, 19° and 29°^32^) are observed in XRD pattern. This can be explained by the sublimation of non-reacted MAI due to the thermal energy provided by the BiI_3_ molecules and the heat released during the condensation of both precursors. Thus, the simultaneous deposition has a self-limitation for MAI incorporation, leading to the formation of stoichiometric MBI perovskites. Based on the absorption measurements, the optimal ratio of MAI and BiI_3_ is determined to 5. As a consequence, this ratio is used for further deposition experiments.

**Figure 6:**
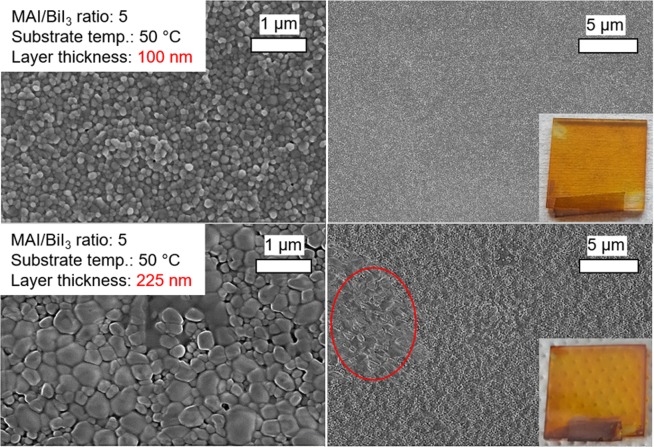
SEM images of MBI films deposited at 50 °C substrate and layer thicknesses of 100 nm and 225 nm. The insets show the macroscopic appearances (photography, 2.5 cm × 2.5 cm).

We decreased the temperature of the substrate from 88 °C to 50 °C to analyze the impact of thermal energy during film formation and its influence on layer morphology. MBI films with layer thicknesses of 100 nm and 225 nm were fabricated at 50 °C substrate temperature using the optimal MAI/BiI_3_ ratio of 5. The SEM images (Fig. 6, upper images) of the layer with a thickness of 100 nm exhibit the same average grain size of ca. 100 nm, but a significantly higher density of rounded grains compared to those film processed at 88 °C substrate temperature and with the same MAI/BiI_3_ ratio of 5 (Fig. 3, center, layer thickness 130 nm).

**Figure 7:**
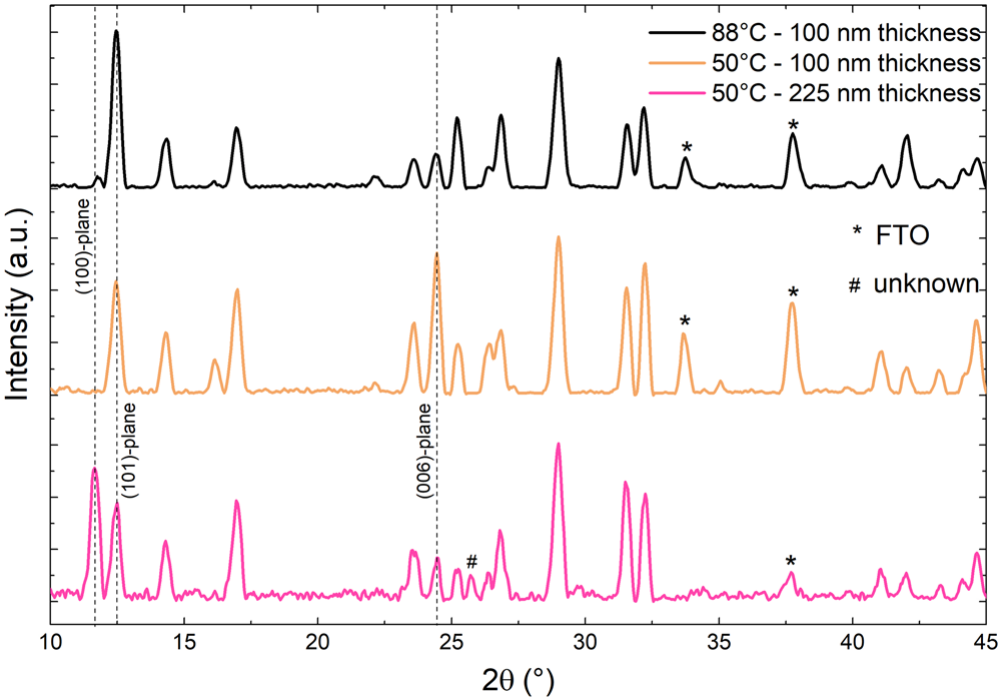
XRD patterns of MBI grown at 50 °C substrate temperature with different layer thicknesses of 100 nm and 225 nm compared to MBI film deposited at 88 °C with 100 nm layer thickness.

The higher layer thickness of 225 nm is beneficial for solar cell application^22^ and was deposited in order to support the formation of larger grains^33^, which are desired to obtain higher photocurrents in perovskite solar cells^34,35^. The SEM images in Fig. 5 (lower images) exhibit larger rounded crystallites with sizes up to 500 nm. Regions with even bigger, micrometer-sized grains can be seen in lower magnification (marked with a red circle). Hence, reducing the substrate temperature to 50 °C leads to rounded grains, the sizes of which appear to scale with the layer thickness. As can be seen from SEM cross-section (Fig. S1), the larger crystallites are present through the entire perovskite layer confirming the continuous growth of initially formed smaller crystallites. However, reducing the substrate temperature leads to diverging crystal orientations, as can be seen from XRD measurements (depicted in Fig. 7).

The XRD spectrum of the film grown at 88 °C substrate temperature (layer thickness 100 nm) matches the calculated pattern and serves as the reference. The XRD measurement of the sample with the same layer thickness, but deposited at 50 °C shows a reduced intensity of the main reflex at 12.5° ((101) plane) and a higher relative intensity at 24.4° ((006) plane). This is indicating a preferred orientation with its (006) plane parallel to the substrate, which was also observed in literature for MBI layers^30^. The analysis of the sample grown at 50 °C with a layer thickness of 225 nm reveals that the spectrum also deviates from the ideal pattern, exhibiting a preferential orientation of the (100) plane (11.7°). However, there is no evidence which MBI crystallite orientation is more suitable for PV application. So far, we could not assign the peak at 25.8° (marked with #) to any material or perovskite phase clearly. This reflex could refer to residual BiI_3_ as pure BiI_3_ films exhibit a peak at 25.5° belonging to its (021) plane. As expected, the film shows reduced intensity of the FTO peaks due to the higher perovskite layer thickness (225 nm).

For layers processed at 50 °C substrate temperature, we found an additional annealing step (100 °C for 60 min on a hot-plate) to be beneficial to achieve the calculated crystal orientation (XRD pattern shown in the supporting material, Fig. S2). Thus, the lower substrate temperature effects preferred orientations and hinders uniformly distributed crystal orientations, most likely due to limited diffusion.

**Figure 8:**
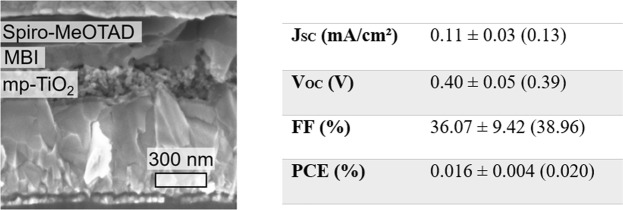
SEM cross-section image (left) and PV characteristics (AM1.5, 100 mW/cm²) of MBI perovskite solar cell (right), fabricated using MBI layer deposited by CVD (50 °C substrate temperature, MAI/BiI_3_ ratio of 5 and 225 nm layer thickness). Average and standard deviation based on 5 devices. Characteristics of the best performing solar cell are shown in parentheses.

Solution-processed MBI typically suffers from not entirely closed films due to needle-shaped crystals orientated parallel to the substrate. However, those films penetrate into the mp-TiO_2_ matrix and feature reasonable device efficiencies. As can be seen from the SEM cross-section (Fig. 8), the CVD-processed MBI films are characterized by a weak penetration into the mp-TiO_2_ matrix. Especially for MBI with short exciton diffusion length (<50 nm^36^), the reduced interface area may hamper the extraction of charge carriers. Thus, all fabricated solar cells feature limited device performance so far. The PV characteristics J_SC_ (0.1 mA/cm²), V_OC_ (0.4 V) and PCE (0.02%) are significantly lower compared to values from MBI layers, which were processed from solution with the same layer thickness and employed in the identical device architecture (J_SC_ > 0.5 mA/cm², V_OC_ > 0.7 V and PCE of 0.17%)^22^. However, the comparison of the FF exhibits no significant differences. IV measurements of the best performing device are shown in Fig. S3, possessing a quasi-hysteresis-free characteristic. An increase of device performance is conceivable by optimizing the process conditions for enhanced penetration of MBI into the mp-TiO_2_ matrix. Furthermore, the application of CVD-processed MBI in inverted device structures or planar architectures (without mp-TiO_2_) will be examined.

## Conclusions

In this work, we demonstrate the first showerhead-based CVD of MBI layers, enabling large-area production. We determined the required ratio of precursors to form perovskites and verified the synthesis of MBI by optical absorption and XRD measurements. We found that the simultaneous deposition has a self-limitation for MAI incorporation, leading to the formation of stoichiometric MBI perovskites. Substrate temperature plays an important role for the crystal structure. Reducing the temperature from 88 °C to 50 °C provides rounded crystallites and different preferential orientations observed in XRD patterns. Moreover, we demonstrate the control of the crystallite size by adjusting the layer thickness. Further optimization of the deposition process (e.g. a tailored layer formation step to ensure crystallite growth or additional thermal annealing) could provide larger grains. CVD MBI was implemented in perovskite solar cells. The devices feature limited device performance due to weak penetration of MBI into mp-TiO_2_ matrix. Future experiments should intend improved contact between the bottom electrode and the perovskite layer. The presented study highlights showerhead-based CVD as a promising deposition method to enable large-area fabrication of environmentally benign PV devices. Furthermore, CVD could be the method of choice for Pb-free multi-cation perovskites with well controlled composition and morphology.

## Supplementary information

Supplementary Information

## Data Availability

The data of this study are available from the corresponding author upon reasonable request.
