# Supplementary material for: Chemical Vapor Deposition of Organic-Inorganic Bismuth-Based Perovskite Films for Solar Cell Application
Source: Sci Rep. 2019 Jul 5;9:9774. doi: 10.1038/s41598-019-46199-4 (PMC6611780; doi:10.1038/s41598-019-46199-4)
Supplement: Supplementary file 1 — Supplementary Information [file 41598_2019_46199_MOESM1_ESM.docx]

Supplementary Information

Chemical Vapor Deposition of Organic-Inorganic Bismuth-Based Perovskite Films for Solar Cell Application

S. Sanders^1,*^, D. Stümmler^1^, P. Pfeiffer^1^, N. Ackermann^1^, G. Simkus^1,2^, M. Heuken^1,2^, P. K. Baumann^3^, A. Vescan^1^ and H. Kalisch^1^

^1^ Compound Semiconductor Technology, RWTH Aachen University, Sommerfeldstr. 18, 52074 Aachen, Germany

^2^ AIXTRON SE, Dornkaulstr. 2, 52134 Herzogenrath, Germany

^3^ APEVA SE, Dornkaulstr. 2, 52134 Herzogenrath, Germany

*Corresponding author: sanders@cst.rwth-aachen.de


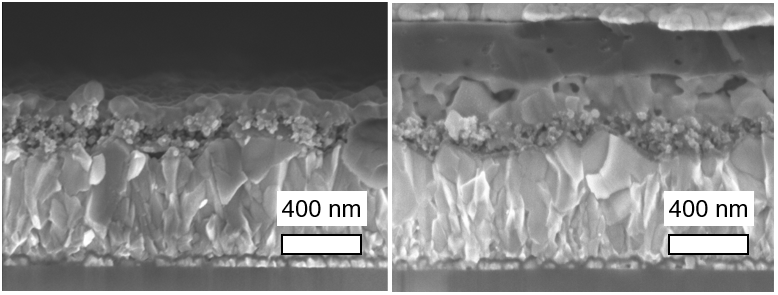


**Figure S1.** SEM cross-section images of MBI layers, deposited by CVD at 50 °C substrate temperature and with a layer thickness of 100 nm (left) and 225 nm (right, also with Spiro-MeOTAD and Au on top).

**Figure S2.** XRD patterns of MBI layer with a thickness of 180 nm grown at 50 °C substrate temperature with and without additional annealing step (60 min at 100 °C on a hot plate). Without annealing the sample shows a high intensity at 26.4° ((2 -1 4) plane). This is indicating a preferential orientation with its (2 -1 4) plane parallel to the substrate. After annealing, the sample exhibits a reduced preferential orientation of the (2 -1 4) plane. Thus, the annealing step effects more uniformly distributed crystal orientations.

**Figure S3.** IV measurements (AM1.5 100 mW/cm2) of the best performing CVD-MBI-based perovskite solar cell, executed in both scan directions.
